# Joint Optimization for Mobile Edge Computing-Enabled Blockchain Systems: A Deep Reinforcement Learning Approach

**DOI:** 10.3390/s22093217

**Published:** 2022-04-22

**Authors:** Zhuoer Hu, Hui Gao, Taotao Wang, Daoqi Han, Yueming Lu

**Affiliations:** 1 Key Laboratory of Trustworthy Distributed Computing and Service, Ministry of Education, Beijing University of Posts and Telecommunications, Beijing 100876, China; zhuoer_hu@bupt.edu.cn (Z.H.); handq@bupt.edu.cn (D.H.); ymlu@bupt.edu.cn (Y.L.); 2 College of Electronics and Information Engineering, Shenzhen University, Shenzhen 518060, China; ttwang@szu.edu.cn

**Keywords:** blockchain, mobile edge computing, computation offloading, deep deterministic policy gradient (DDPG)

## Abstract

A mobile edge computing (MEC)-enabled blockchain system is proposed in this study for secure data storage and sharing in internet of things (IoT) networks, with the MEC acting as an overlay system to provide dynamic computation offloading services. Considering latency-critical, resource-limited, and dynamic IoT scenarios, an adaptive system resource allocation and computation offloading scheme is designed to optimize the scalability performance for MEC-enabled blockchain systems, wherein the scalability is quantified as MEC computational efficiency and blockchain system throughput. Specifically, we jointly optimize the computation offloading policy and block generation strategy to maximize the scalability of MEC-enabled blockchain systems and meanwhile guarantee data security and system efficiency. In contrast to existing works that ignore frequent user movement and dynamic task requirements in IoT networks, the joint performance optimization scheme is formulated as a Markov decision process (MDP). Furthermore, we design a deep deterministic policy gradient (DDPG)-based algorithm to solve the MDP problem and define the multiple and variable number of consecutive time slots as a decision epoch to conduct model training. Specifically, DDPG can solve an MDP problem with a continuous action space and it only requires a straightforward actor–critic architecture, making it suitable for tackling the dynamics and complexity of the MEC-enabled blockchain system. As demonstrated by simulations, the proposed scheme can achieve performance improvements over the deep Q network (DQN)-based scheme and some other greedy schemes in terms of long-term transactional throughput.

## 1. Introduction

With the wide utilization of intelligent mobile devices in the fifth-generation (5G) era and the advances in wireless communication techniques in the forthcoming sixth-generation (6G) communication systems [1], the internet of things (IoT) is increasingly attracting attention from both academia and industry as a versatile technology [2,3]. Nowadays, more and more intelligent devices get access to IoT networks, where each object with identifying, sensing, networking, and processing capabilities can communicate with other nodes. Such ubiquitous interconnections generate massive data to be stored, processed, and analyzed [4,5].

Recent advances in information and communication technologies are accelerating the IoT’s transition to the 6G era. As a result, new IoT infrastructures and data processing architectures are under construction. Currently, a majority of IoT applications depend on centralized cloud servers to store data and process tasks [6], which necessitates that the third parties owning the cloud servers are quite trustworthy, otherwise the user data may be exposed to security concerns [7]. Furthermore, centralized cloud-based applications introduce delay and privacy issues [8,9]. Therefore, demands for data security, processing efficiency, and low operational cost are overgrowing in IoT scenarios [10]. Blockchain [11], as a decentralized data storage technology, can guarantee that each transaction record is immutable through an encryption algorithm and distributed structure. As a result, blockchain has been presented as a promising technique for enhancing the security and efficiency of data storing/fetching in IoT networks, which can realize tamper resistance and data availability for IoT networks in a decentralized manner [12,13,14].

In the current IoT architecture, blockchain technology has been extensively investigated [15]. Kang et al. have proposed a blockchain-enabled internet of vehicles framework based on an upgraded delegated proof-of-stake (DPoS) consensus mechanism rather than the native proof-of-work (PoW) or proof-of-stake (PoS) mechanism to improve the security of vehicle data sharing [16]. Fan et al. presented a blockchain-based strategy for resolving the security issue associated with time synchronization in IoT networks [17]. W. Li et al. proposed a data security strategy based on blockchain technology for intelligent applications in 6G systems [18]. However, these schemes focus on the security of block verification and block generation while lacking the consideration of emerging applications and services in IoT networks that require high computational workloads and low latency. Most existing blockchain-enabled frameworks cannot meet the demand for high transactional throughput in IoT scenarios with compute-intensive and real-time applications and services. Therefore, service-oriented blockchain-enabled frameworks are called for to meet the transactional throughput demands of current and next-generation IoT networks.

New IoT services and applications, such as mobile multimedia, visual sensors, smart grids, and intelligent vehicles, are computationally intensive and sensitive to latency, challenging the blockchain-enabled IoT framework design. Conventional cloud computing systems are confronted with the problems of long latency and overload problems, hindering the blockchain deployment in IoT networks. To address the above challenges, the mobile edge computing (MEC) [19] technique is considered a potential option. Because an MEC system is deployed at the edge of IoT networks and near the access network, it is capable of bridging the divide between the limited resources in the proximity of users and the ever-increasing computational demand of IoT applications [20]. Therefore, MEC technology can facilitate the development of low-latency, scalable, and blockchain-enabled IoT networks. Recently, several studies [21,22] have been proposed to enhance data security and transactional throughput for IoT by integrating blockchain technology and MEC technology. However, most existing schemes focus only on the computational offloading strategy of MEC or the working mechanism of blockchain and lack a comprehensive and specific analysis of the MEC-enabled blockchain system. Therefore, they fail to achieve a joint performance optimization, which leaves room to improve.

The efficient deployment and optimization of MEC-enabled blockchain systems in IoT networks are challenging from several perspectives: trade-off between latency and security, joint optimization, and dynamic continuous domain-based optimization. (1) *Trade-off between latency and security*: the blockchain-enabled IoT network is confronted with latency-sensitive challenges and thus requires an efficient blockchain mechanism without compromising security. Additionally, due to system resource limitations [23] in IoT networks, the collaborative design of an efficient resource allocation policy and a lightweight blockchain consensus mechanism at the edge of wireless networks is challenging. (2) *Joint optimization*: the optimization problem formulation in most existing studies [21,22] considers the blockchain system and MEC system separately, ignoring the coupling relationship between the blockchain transactional throughput and the MEC computation rate. Due to the lack of joint optimization consideration, the performance in existing studies can be improved. (3) *Dynamic continuous domain-based optimization*: the IoT services arrive in complicated stochastic patterns, and most computation tasks of IoT services have sensitive latency requirements, which also pose significant challenges to the joint optimization of MEC-enabled blockchain systems. Additionally, the parameters of MEC computation offloading policy or blockchain optimization strategy are continuous domain variables, while existing works [21,22] simplify them to discrete variables. Therefore, we resort to continuous domain-based DRL to realize dynamic and continuous joint control of computation resource allocation and block generation.

To address the aforementioned issues, we present a DRL-based joint performance optimization framework for MEC-enabled blockchain systems in IoT networks, which aims to improve the scalability/throughput while guaranteeing data security and transaction processing efficiency. In particular, each IoT node can offload a portion of computation tasks to MEC servers for efficient data processing, wherein the computation tasks include the IoT application tasks and the tasks for block generation and reaching consensus. Meanwhile, blockchain technology is adopted for secure data storage and sharing inside this framework, with a consensus mechanism based on practical Byzantine fault tolerance (PBFT) and DPoS [24] being adopted. Furthermore, the performance optimization of MEC and blockchain system is jointly formulated as a Markov decision process (MDP) problem, where state transitions mainly depend on changes in time-varying factors such as the impact of user movement on wireless transmissions, node workload, etc, which are unknown a priori. Moreover, the action is selected based on continuous space. As a result, conventional math models are ineffective in solving the MDP problem. To address this MDP problem, a novel DRL-based algorithm with continuous action space is developed. It shows superiority in tackling dynamic and complicated joint optimization problems. The primary contributions of this paper are listed as follows:

(1) A novel MEC-enabled blockchain framework in IoT networks is developed, considering the latency and scalability issues that arose from the throughput requirements of future wireless networks and blockchain systems. We analyze MEC computation efficiency and critical performance indicators of blockchain, i.e., decentralization, latency, throughput, and adversarial fraction, which can guide the joint optimization of the framework.
(2) A novel MEC and blockchain joint optimization algorithm is developed for maximizing the computational efficiency of MEC and the transaction throughput of blockchain systems, which is formulated as an MDP problem. In contrast to most existing research [15,16,17] in which the modeling and optimization of MEC and blockchain systems are carried out independently, the block interval, block size, data transaction throughput, power allocation for local execution and task offloading, latency, and security constraints are jointly considered in the proposed algorithm. Therefore, we propose a more comprehensive scheme and address the blockchain deployment challenges in IoT scenarios.
(3) The MDP problem is solved using a deep deterministic policy gradient (DDPG)-based learning algorithm to tackle the dynamic and large-dimensional properties of IoT networks that are intractable using classic learning approaches such as Q-learning [25]. In particular, the DDPG-based algorithm enables the joint resource allocation for MEC and blockchain systems in a continuous domain so as to solve the MDP problem with better convergence.
(4) Extensive simulation findings demonstrate that the presented performance optimization framework has the capacity to enhance the transaction processing efficiency of MEC-enabled blockchain networks significantly. The superiority of the DDPG-based algorithm over the deep Q network (DQN)-based algorithm [26] and other conventional schemes is verified.

The remainder of this paper is structured as follows. Section 2 introduces the related works. Section 3 provides the preliminaries of blockchain technology and a basic introduction of the consensus protocol based on DPoS and PBFT. Section 4 describes the system model. In Section 5, the DDPG-based joint performance optimization framework is proposed, wherein the joint optimization problem is formulated and solved using a DDPG-based approach. Section 6 evaluates the proposed algorithm in detail and discusses the simulation results. At last, Section 7 summarizes this paper and looks forward to future work.

## 2. Related Works

In this section, we first introduce the existing works on blockchain-enabled IoT networks. Then, existing works which utilize the MEC technology to improve the transaction throughput of blockchain-enabled IoT networks are introduced, and the main distinguishments between our proposed scheme and closely related works are discussed.

### 2.1. Blockchain-Enabled IoT Networks

Due to the properties of blockchain technology such as stability, privacy protection, and security, integrating blockchain technology into IoT systems for supporting IoT future development has received considerable attention recently [27]. Yang et al. present a blockchain-based system for IoT devices and a tailored smart contract to enable the holistic transactive energy management [28]. Nguyen et al. design a blockchain-based model for IoT data trading which ensures security and privacy [29]. Although blockchain provides security and privacy benefits for IoT networks, there remains a barrier to its adoption in IoT networks due to the resource constraints of IoT devices.

### 2.2. MEC-Enabled Blockchain for IoT Networks

MEC technology has been widely utilized to perform massive, parallel, and complex computations [30]. The MEC system enables resource-constrained IoT devices to offload computing tasks to edge MEC servers, therefore resolving the resource-intensive issues that blockchain-enabled IoT networks confront. There are some works that use the MEC technology to improve the transaction throughput of blockchain-enabled IoT networks, which are closely related to our study. Zhao et al. proposes a computation resource allocation strategy of a public blockchain network in MEC systems [21], which is designed for a one-time slot and would involve a huge computation cost for long-term performance optimization that edge devices cannot afford. Nguyen et al. presents a secure deep reinforcement learning (DRL)-based computation offloading approach and a trustworthy blockchain access control mechanism for the mobile blockchain-based IoT networks [22]. Qiu et al. develops an adaptive genetic algorithm (AGA)-based computation offloading scheme for MEC-enabled blockchain systems [31]. Guo et al. proposes a resource allocation and block generation scheme based on a double-dueling DQN algorithm [32]. However, both [22,31] perform the optimization of blockchain and MEC systems independently, resulting in inferior performance. Additionally, both [22,32] are discrete action space-based and incapable of handling continuous action cases. Therefore, we develop a DDPG-based joint performance optimization algorithm for blockchain and MEC systems while considering the decentralization, security, latency, and power consumption constraints.

In summary, Table 1 shows distinguishments between our proposed scheme and some existing research. Specifically, for the ‘Blockchain Agent’ column, we can find that the deployment of the blockchain agent in the proposed scheme differs from [31,32], which results in distinct system model designs. For the ‘State Space Design’ column, A considers system offloading cost, system computation resources, and system bandwidth resources; B considers task queue length, computation resources of users, network identification, and available server resources; C considers channel condition between users and BSs, channel condition between different BSs, computation resources of BSs, primary node; D considers channel condition between users and BSs, computation resources of users, task buffer of users, and stake distribution. All in all, the blockchain agent deployment and state space design in this paper differ from existing ML-based schemes and therefore leads to different system model and problem formulation.

## 3. Preliminaries on Blockchain

This section will begin with an overview of blockchain technology, followed by an introduction to the fundamentals of the consensus protocol based on DPoS and PBFT.

### 3.1. Overview of Blockchain

A blockchain is an encrypted and distributed database that maintains an ordered collection of transaction records. Blockchain has many security performance advantages over traditional centralized databases [33]. Firstly, the primary objective of blockchain technology is to prevent the database’s data from being maliciously tampered with or stolen. Furthermore, the decentralized nature of the blockchain system distributes and stores blocks that contain transaction records across a large number of network nodes during the block generation process. Due to the fact that the block generation process involves a large number of network nodes, data transactions are only recorded on a block after it has been confirmed by other nodes. Therefore, data storage is not dependent on a single centralized database. As a result, blockchain technology outperforms traditional centralized databases in terms of reliability and resistance to the single point of failure attacks.

**Figure 1:**
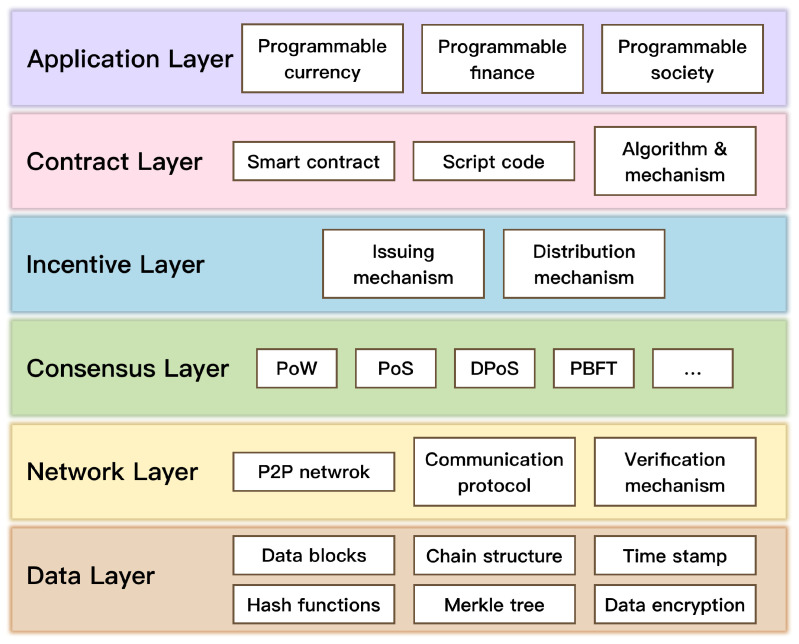
General architecture of blockchain.

The general architecture of blockchain technology consists of six layers [34] as shown in Figure 1. A blockchain-enabled system is comprised of the following components: an encryption method, peer-to-peer (P2P) transmission, a consensus protocol, a distributed ledger, a smart contract, and the application scenario [35]. In particular, the consensus protocol is a critical component of the blockchain. A blockchain system based on the consensus protocol can validate data transaction records without the involvement of a trusted third party. In the following, the consensus protocol adopted in this study will be introduced.

### 3.2. Consensus Protocol Based on DPoS and PBFT

This subsection focuses on the consensus protocol, which is an important component of the blockchain system. Different blockchain systems handle the consensus problem by adopting different protocols that require validators to demonstrate their neutrality [36]. The most widely used consensus protocols include PoW, PoS, DPoS, and PBFT. The DPoS protocol, derived from the PoS protocol, involves the voting and electing mechanisms so as to classify participating nodes as different roles to perform different functions in the consensus process. In comparison to the PoW and PoS protocols, the DPoS protocol enables faster block validation [37].

PBFT is a widely used and well-studied consensus algorithm, and the whole consensus process of it consists of five steps: *Request*, *Pre-prepare*, *Prepare*, *Commit*, and *Reply* [38]. Existing research has demonstrated the effectiveness of the PBFT consensus algorithm for deployment on resource-constrained IoT devices [39]. To further adapt to large-scale IoT networks with a huge number of device nodes, the consensus protocol adopted in this study is a combination of PBFT and DPoS, called the BFT-DPoS consensus protocol, and it inherits the delegate election mechanism in DPoS [24]. In contrast to PBFT, the BFT-DPoS consensus protocol only involves a subset of delegate nodes (i.e., validation nodes) rather than all device nodes during the voting process. For example, there is one client, one primary node, and three other validation nodes. Once the consensus is triggered, the primary node broadcasts a *Pre-prepare* proposal to other validation nodes. During *Prepare* and *Commit* phases, all validation nodes exchange messages to check the reliability and validity of received messages. A node steps into the subsequent phase after receiving more than $\frac{2}{3}$ acknowledgments that include its own.

According to the BFT-DPoS consensus protocol, each general node in the framework can store/fetch transaction data into/from the blockchain system. At the same time, only a part of the nodes can be elected as validation nodes in terms of the number of stakes and available computation resources each node holds. Therefore, when one new block is formed, the new block proposal needs to be broadcasted to other validation nodes. Only if this block has been verified and most of the validation nodes have reached consensus, will it be appended to the main blockchain. Additionally, the communication complexity of PBFT increases exponentially with the number of participating nodes, so there is a trade-off between the communication complexity and security [40].

## 4. System Model

**Figure 2:**
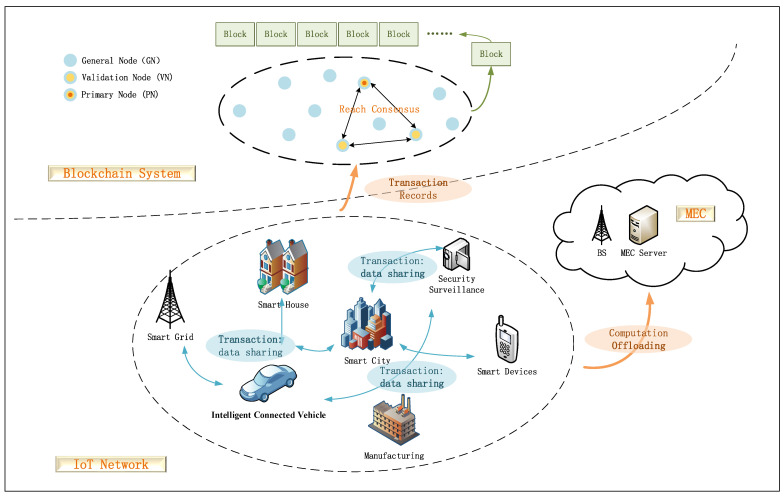
MEC-enabled blockchain system in the heterogeneous IoT networks.

In this section, the system model adopted in this work is introduced. As illustrated in Figure 2, we propose an MEC-enabled blockchain framework for IoT networks, which comprises three parts, i.e., the IoT network including various smart devices, an MEC system including the BS and MEC servers, and a blockchain system based on the DPoS mechanism.

In the IoT network, smart devices, e.g., vehicles, cell phones, security surveillance, etc., collect some ambient data that must be securely stored/processed or shared with other IoT smart devices. As a result, we consider two types of data transactions among smart devices: (1) data storage/processing and (2) data sharing, both of which are stored in the blockchain system for distributed and secure data storage/retrieval. The nodes in the blockchain system are classified into three categories: (1) general nodes (GNs) that consist of all IoT devices, (2) validation nodes (VNs) that are selected out of GNs based on a specific stake distribution according to the DPoS mechanism, and (3) one primary node (PN) that is selected from VNs and authorized to produce blocks at a specific decision epoch. The blockchain system is mainly responsible for the secure storage/retrieval of transaction data from the IoT network. To achieve this goal, the blockchain system must generate blocks and reach consensus, where GNs receive/transmit transaction data from/to other nodes, VNs conduct the blockchain consensus process, and the PN is authorized to generate blocks within a specific time period. Moreover, the MEC system is responsible for sharing the computational pressure in the IoT network and blockchain system to achieve efficient data processing and blockchain consensus. In the following subsections, we will detail the network model, MEC model, and blockchain model, respectively. The notations used in this paper are summarized in Table 2.

### 4.1. Network Model

In this paper, we assume that the blockchain system has *M* GNs denoted by $\Phi_{G}$ and *K* VNs. For each IoT node $m\in \Phi_{G}$, it conducts data storage/processing and data sharing within the IoT network. Meanwhile, it acts as a part of the blockchain system. Specifically, *K* VNs, denoted by $\Phi_{V}$, $\Phi_{V}\subseteq \Phi_{G}$, are selected out of $\Phi_{G}$ in terms of particular rules [41]. These VNs are responsible for collecting, validating, and packaging the transactions generated by smart devices into a block. Furthermore, this new block is appended to the blockchain after the PN broadcasts the block proposal to other VNs and a consensus is reached.

**Figure 3:**
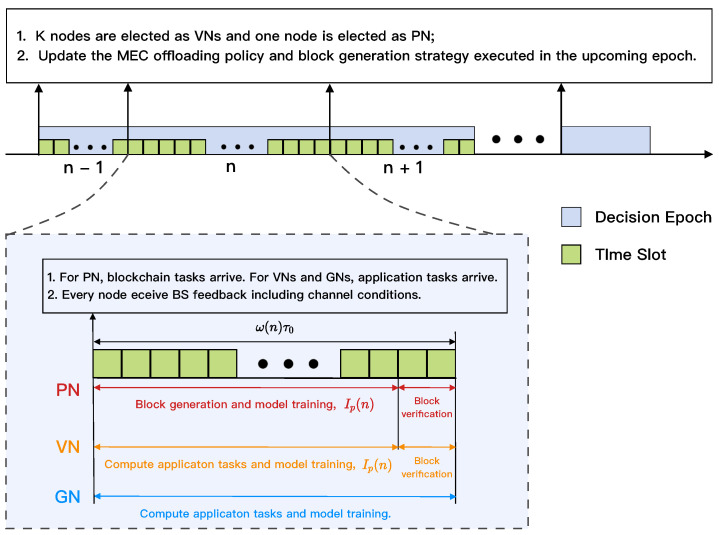
The relationship between time slot τ and decision epoch *n*.

As shown in Figure 3, the MEC-enabled blockchain framework is implemented using a discrete-time model in which time is partitioned into multiple decision epochs, and the *n*-th epoch, $n\in \{0,1,\ldots ,N_{max}\}$, has $L_{n}$ basic time slots which has an identical duration $\tau_{0}$ and is indexed by τ∈{0,1,…}. Thus, each decision epoch *n* has a dynamic duration $L_{n}\tau_{0}$, where $L_{n}\in \left\{1,2,\ldots ,\dot{L_{n}}\right\}$ varies for each decision epoch *n* and is determined by the block interval of PN $I_{P}\left(n\right)$. For each decision epoch t∈T, the wireless channel condition, task arrival, and power allocation policy of each GN are varied. Therefore, aiming to balance data processing efficiency, security, and average energy consumption, each VN needs to determine the block size, block interval, and task offloading policy in each epoch. Assume that the PN produces a new block with block size $S^{B}\left(n\right)$ and block interval $I_{P}\left(n\right)$ in turns within the *n*-th epoch. Specifically, block size $S^{B}\left(n\right)$ indicates the number of bits included in a new block produced by the PN at the end of epoch *n*, while block interval $I_{P}\left(n\right)$ indicates the time required for the VN to generate a new block in each epoch *n*.

In the proposed framework, we analyze a 5G macro-cell base station (BS) having $N_{a}$ antennas that handle the uplink communications of numerous IoT nodes with a single antenna using linear zero-forcing (ZF) detection [42], which is simple and efficient [43]. In this study, we assume that the number of antennas at the BS exceeds the number of mobile nodes, i.e., $N_{a}>M$. For each time slot τ, we denote the channel vector of each GN *m* as $h_{m}\left(\tau \right)\in C^{N_{a}\times 1}$, and therefore the nearest BS’s received signal can be represented by:

(1) $$y\left(\tau \right)=\sum_{m=1}^{M}h_{m}\left(\tau \right)\sqrt{p_{o,m}\left(\tau \right)}s_{m}\left(\tau \right)+n_{G}\left(\tau \right),$$

where $p_{o,m}\left(\tau \right)\in \left[0,{\dot{P}}_{o}\right]$ denotes the uplink power for GN *m* to offload transaction tasks with the upper bound ${\dot{P}}_{o}$, $s_{m}\left(\tau \right)$ denotes the complex data symbol. In addition, $n_{G}\left(\tau \right)∼CN_{┤}\left(0,\sigma_{R}^{2}I_{N_{a}}\right)$ is a noise vector where $\sigma_{R}^{2}$ denotes the variance and $I_{N_{a}}$ is a $N_{a}\times N_{a}$ identity matrix. Furthermore, we adopt the following Markov block fading auto-regressive model [44] to define the temporal relation between decision epochs and movement for each GN *m*:

(2) $$h_{m}\left(\tau +1\right)=\rho_{m}h_{m}\left(\tau \right)+\sqrt{1-\rho_{m}^{2}}e\left(\tau +1\right),$$

where $\rho_{m}=J_{0}\left(2\pi f_{m}^{d}\tau_{0}\right)$ denotes the normalization of correlation function between time slots τ+1 and τ in terms of Jake’s fading spectrum, and the error vector $e\left(\tau \right)∼CN\left(0,\sigma_{R}^{2}I_{N_{a}}\right)$ is complex Gaussian and independent identically distributed with $h_{m}\left(\tau \right)$. It is worth noting that $f_{m}^{d}$ and $J_{0}\left(\cdot \right)$ denote the Doppler frequency of GN *m* and first-order Bessel function, respectively.

The N×M channel matrix between the considered 5G macro-cell BS and *M* GNs is represented by $H\left(\tau \right)=\left[h_{1}\left(\tau \right),h_{2}\left(\tau \right),\ldots ,h_{M}\left(\tau \right)\right]$. The linear zero-forcing detection is derived by $H^{†}\left(\tau \right)={\left(H^{H}\left(\tau \right)H\left(\tau \right)\right)}^{-1}H^{H}\left(\tau \right)$. After applying the ZF detector, each node’s signal to interference-plus-noise ratio (SINR) is calculated by [43]:

(3) $$\gamma_{m}\left(\tau \right)=\frac{p_{o,m}\left(\tau \right)}{\sigma_{R}^{2}{\left[{\left(H^{H}\left(\tau \right)H\left(\tau \right)\right)}^{-1}\right]}_{mm}},$$

where ${\left[I\right]}_{m_{1}m_{2}}$ denotes the $\left(m_{1},m_{2}\right)$-th item in matrix I.

For each decision epoch *n*, we assume that the channel condition and the power allocation (i.e., $p_{o,m}\left(n\right)$ and $p_{l,m}\left(n\right)$) is consistent throughout one decision epoch and updated at the first time slot of each epoch.

### 4.2. MEC Model

In this subsection, we will show how each GN *m* makes use of an adaptive compute offloading policy to support blockchain-enabled IoT networks. $a_{m}\left(n\right)$ (bit) is the number of computing tasks during the decision epoch *n*, which is assumed to be processed since decision epoch n+1. In addition, we consider that $a_{m}\left(n\right)$ is independent and identically distributed throughout different decision epochs and there is an average task-arrival rate $\lambda_{m}=E\left[a_{m}\left(n\right)\right]$ based on Poisson distribution. In general, $a_{m}\left(n\right)$ is considered as ordinary application tasks, while for the node elected as PN at decision epoch *n* we consider $a_{P}\left(n\right)$ as block-generation tasks. Furthermore, the processing of computation tasks is considered fine-grained [45]. Therefore, within the *n*-th decision epoch, $D_{l,m}\left(n\right)=L_{n}d_{l,m}\left(\tau \right)$ denotes partial bits of computation tasks that are allocated to be processed locally, while $D_{o,m}\left(n\right)=L_{n}d_{o,m}\left(\tau \right)$ represents some other bits that are transferred to and processed by the edge server. We denote $U_{m}\left(n\right)$ as the queue length of the GN *m*’s task buffer at decision epoch *n*’s commencement, and then $U_{m}\left(n+1\right)$ can be expressed by:

(4) $$\begin{array}{cc} U_{m}\left(n+1\right)= & {\left[U_{m}\left(n\right)-\left(D_{l,m}\left(n\right)+D_{o,m}\left(n\right)\right)\right]}^{+}+a_{m}\left(n\right), \end{array}$$

where ${\left[x\right]}^{+}=max\left(0,x\right)$ and $U_{m}\left(0\right)=0$.

#### 4.2.1. Local Computation

This section demonstrates the number of data bits handled locally in relation to the power allocated for local computing $p_{l,m}\left(\tau \right)\in \left[0,{\dot{P}}_{l}\right]$. By chip voltage adjustment based on the dynamic voltage and frequency scaling technology [46], the central processing unit (CPU) frequency (Hz) scheduled for the time slot τ is expressed as:

(5) $$f_{m}\left(\tau \right)=\sqrt[{3}]{p_{l,m}\left(\tau \right)/\iota },$$

in which ι denotes the effective switching capacitance of the chip, which varies according to its architecture [46]. Additionally, we have $0\leq f_{m}\left(\tau \right)\leq F_{m}$ and $F_{m}=\sqrt[{3}]{{\dot{P}}_{l}/\iota }$, which is the highest permitted CPU frequency of GN *m* depending on system capability. Consequently, the number of locally processed bits during the time slot τ is calculated by multiplying the time (s) by the computation rate of the device CPU (bit/s). Specifically, the computation rate of the device CPU (bits/s) is derived by multiplying the device CPU frequency (cycles/s) by the number of task bits that the CPU can process per cycle (bits/cycle). That is,

(6) $$d_{l,m}\left(\tau \right)=\tau_{0}f_{m}\left(\tau \right)C_{m}^{-1},$$

where $C_{m}$ (cycles/bit) denotes the number of CPU cycles necessary for GN *m* to compute one data bit and it is measured and determined with offline measurement [47].

#### 4.2.2. Edge Computation

To start with, we assume that the MEC server can handle different computation tasks with a minimal processing delay due to adequate computational resources such as a high-frequency multicore CPU. In addition, as a result of the small-sized computation output, it can be assumed that the feedback delay between BS and node can be ignored. Based on (3) and with the uplink communication power $p_{o,m}\left(\tau \right)$, the number of task bits offloaded by GN *m* within the time slot τ is calculated by:

(7) $$d_{o,m}\left(\tau \right)=\tau_{0}Wlog_{2}\left(1+\gamma_{m}\left(\tau \right)\right),$$

where *W* denotes the system bandwidth.

Therefore, the computation rate (bits/s) of GN *m* during time slot τ is calculated by:

(8) $$\nu_{m}\left(\tau \right)=f_{m}\left(\tau \right)C_{m}^{-1}+Wlog_{2}\left(1+\gamma_{m}\left(\tau \right)\right).$$

According to the MEC computation model, each IoT node with stochastic task arrivals can utilize the nearby MEC server to process the compute-intensive tasks efficiently and adjust the ratio of computation offloading dynamically.

### 4.3. Blockchain System

In this subsection, we present the considered blockchain system. The blockchain system can provide data security and privacy guarantee to the IoT networks as an overlaid system. Each node inside the blockchain system is capable of collecting data transactions from the IoT networks, while only a few with a large number of stakes are elected as VNs for packaging and validating blocks. We assume that $Y\left(n\right)=\{Y_{1}\left(n\right),Y_{2}\left(n\right),\ldots ,Y_{M}\left(n\right)\}$ denotes the set of stakes IoT nodes hold based on the BFT-DPoS consensus algorithm [24] during the *t*-th decision epoch. The stake of each GN is updated when consensus is reached on a new block, and the latest stake distribution is aggregated to the BS. For the start of each decision epoch *n*, the BS distributes the latest stake distribution Y(n) it has recorded to all GNs.

In the following part, the details of the most significant criteria for evaluating the blockchain system performance are described.

#### 4.3.1. Decentralization

To prevent block packaging and verification power from being monopolized, it is necessary to quantify the degree of decentralization to ensure the long-term fairness of the blockchain system [41]. We make use of *Gini  coefficient*, which has been widely used to measure wealth or income inequality [48], evaluate “contrast intensity” [49] and capture “system inequality” [50] in existing works. Focusing on the decentralization of VNs, we take the stake distribution of VNs into account in this study. Therefore, the Gini coefficient of stake distribution can be derived to characterize the decentralization:

(9) $$\begin{array}{c} G\left(Y\left(n\right)\right)=\frac{\sum_{i,j\in \Phi_{V},i\neq j}\left|Y_{i}\left(n\right)-Y_{j}\left(n\right)\right|}{K\sum_{i\in \Phi_{V}}Y_{i}\left(n\right)}. \end{array}$$

G(Y(n)) is within [0,1] in which the extremes 0, 1 denote the perfect uniformity and maximal inequality among stake values, respectively. Hence, to guarantee that VNs’ stake distribution of a blockchain system is decentralized, the Gini coefficient G(Y(n)) should satisfy the constraint:

(10) G(Y(n))≤η,

where we have η∈[0,1]. For simplicity, we assume that G(Y(n))≤η is always satisfied in this work.

#### 4.3.2. Latency Time to Finality and Throughput

The concept of latency time to finality (LTF) is adopted to characterize the latency of the blockchain system, which is the latency that one data transaction record becomes irreversible once it has been committed to the blockchain system [15]. For latency-sensitive applications, it is important to guarantee that the latency is within the user’s tolerance. The LTF $T_{F}\left(n\right)$ including the time cost for block validation $T_{c}\left(n\right)$ and generation $I_{P}\left(n\right)$ is expressed as:

(11) $$T_{F}\left(n\right)=T_{c}\left(n\right)+I_{P}\left(n\right),$$

where $T_{c}\left(n\right)$ denotes the consensus latency which includes the time cost for packet transmission $T_{tr}\left(n\right)$ and packet verification $T_{v}\left(n\right)$ that includes message authentication codes (MACs) generation, request signature, and MACs verification [51]. Note that the latency required for one consensus process in the simulation of this paper is in the order of milliseconds. The majority of real-world mobile IoT devices (e.g., cell phones, smartwatches, etc.) generally have a small displacement within 10 ms. Therefore, we assume that the primary node and consensus nodes usually can provide stable services in one consensus process.

As introduced in Section II, each decision epoch *n* has a dynamic duration $L_{n}\tau_{0}$. We assume that the duration of decision epoch *n* is determined by the LTF $T_{F}\left(n\right)$, and thus we have

(12) $$L_{n}=\left\lceil \frac{T_{F}\left(n\right)}{\tau_{0}}\right\rceil$$

As in [52], the blockchain transaction throughput of the proposed framework within the *n*-th decision epoch is derived by:

(13) $$\Xi \left(n\right)=\frac{\left\lfloor S^{B}\left(n\right)/\chi \right\rfloor }{T_{F}\left(n\right)},$$

where χ denotes average transaction size. $S^{B}\left(n\right)$ is the block size and derived by:

(14) $$S^{B}\left(n\right)=D_{l,P}\left(n\right)+D_{o,P}\left(n\right).$$

where *P* denotes the PN.

#### 4.3.3. Adversarial Fraction

To ensure the blockchain system’s security performance, it is vital to prevent transactions from being unilaterally tampered with or reversed. The adversarial fraction of hashing power that an adversary can control without endangering system security is one kind of fundamental performance measurement of a blockchain system [52]. For the PBFT-based consensus protocols, unambiguous finality can be reached under the assumption that less than a $\frac{1}{3}$ fraction of the nodes are adversarial [24]. Therefore, the following constraint needs to be satisfied:

(15) $$f\leq \left\lfloor \frac{K-1}{3}\right\rfloor ,$$

in which *f* denotes the number of adversarial VNs. For simplicity, in this work, when there are *K* VNs involved in the consensus process, we assume that K≥3f+1. In other words, it is assumed that the above constraint is always met in this work.

## 5. DDPG-Based Performance Optimization Framework

In the following part, we investigate the optimal block generation and computation offloading policies to maximize the transaction throughput of the proposed framework under the constraints of latency and system resources. The joint optimization problem is formulated as an MDP. Moreover, in order to deal with the dynamic and large-dimensional properties of the above-mentioned systems, we develop a DRL-based scheme. Specifically, elements included in the action space (i.e., power allocation and block interval) are continuous variables, which motivates us to employ the DDPG algorithm so as to achieve better performance than the DQN-based approach with a discrete action space [53].

**Figure 4:**
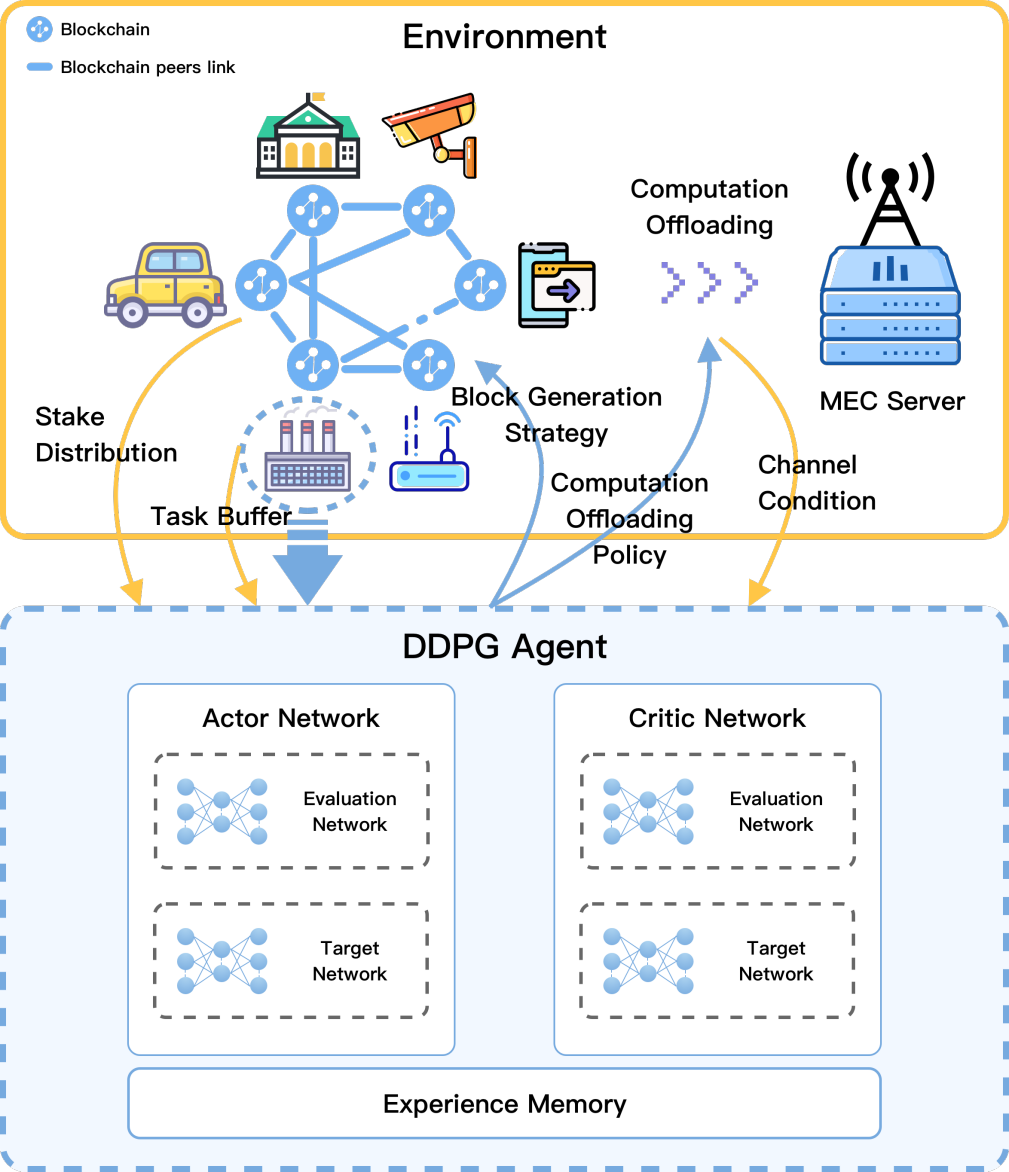
The architecture of the DDPG-based framework.

The architecture of the DDPG-based framework is shown in Figure 4 and the DDPG agent is implemented in each GN. To deploy the framework, we first define the problem formulation and construct the state space, action space, and reward function. Then, we design a DDPG-based algorithm to solve it as follows.

### 5.1. Problem Formulation

Due to the difficulty of obtaining the state transition probabilities and reward values in advance which are related to user mobility, node workload, etc., the optimization problem is constructed as an MDP. As mentioned above, to learn the resource-aware dynamic block generation and computation offloading strategies, we propose maximizing the blockchain transactional throughput and node computation rate while guaranteeing the security and decentralization of data storing/processing with the constraints of computation resources and latency. In other words, each GN *m* needs to solve the following optimization problem in each decision epoch:

(16) $$\begin{array}{cc} \; & P1:\max_{\left\{p_{l,m}\left(n\right),p_{o,m}\left(n\right),I_{P}\left(n\right)\right\}}E\left[\sum_{n=1}^{T_{max}}\left[\omega \Xi \left(n\right)+\left(1-\omega \right)\xi \sum_{m=1}^{M}\nu_{m}\left(n\right)\right]\right]\\ \; & C1:T_{F}\left(n\right)\leq \dot{L_{n}}\times \tau_{0}\\ \; & C2:0\leq p_{o,m}\left(n\right)\leq {\dot{P}}_{o},0\leq p_{l,m}\left(n\right)\leq {\dot{P}}_{l} \end{array}$$

where $\dot{L_{n}}$ is the upper bound of $L_{n}$, ω(0<ω<1) denotes the weight factor for integrating the two objective components, and ξ is a mapping factor which ensures that the blockchain transactional throughput and the MEC total computation rate are of the same order of magnitude.

The constraints C1 and C2 specify latency and power consumption limits, respectively. Note that, for satisfying the latency requirement of IoT applications, it is assumed that each block should be published and validated within $\dot{L_{n}}\left(L_{n}\geq 1\right)$ consecutive time slots.

A decentralized dynamic performance optimization strategy will be learned separately at each node, which determines the block interval and power allocation for both local computing and edge computing, depending on the local observation of the environment. Note that the DDPG-based online learning process is totally model-free, which means this algorithm does not require each node to have prior knowledge of the blockchain and MEC systems.

### 5.2. State Space

It is worth noting that collecting a full observation of the system for all nodes and then distributing them to each node requires high system overheads. Therefore, it is assumed that each node’s state is decided by the observation of the system from its own perspective to avoid high system overheads and make the framework more scalable.

For each decision epoch *n*, the workload of each GN’s task buffer $U_{m}\left(n\right)$ is updated in terms of (4). Meanwhile, GN receives one message from the BS conveying the stake distribution Y(n) and the latest SINR of GN to BS $\gamma_{m}\left(n-1\right)$. In addition, $h_{m}\left(n\right)$ for the forthcoming uplink communication is calculated according to (2). As a result, the state space is defined as:

(17) $$s_{m,n}=\left[U_{m}\left(n\right),Y\left(n\right),\phi_{m}\left(n-1\right),h_{m}\left(n\right)\right],$$

in which the projected power ratio after ZF detection $\phi_{m}\left(n\right)$ is calculated by:

(18) $$\begin{array}{cc} \phi_{m}\left(n\right) & =\frac{\gamma_{m}\left(n\right)\sigma_{R}^{2}}{p_{o,m}\left(n\right){∥h_{m}\left(n\right)∥}^{2}}\\ \; & =\frac{1}{∥h_{m}{\left(n\right)∥}^{2}{\left[{\left(H^{H}\left(n\right)H\left(n\right)\right)}^{-1}\right]}_{mm}}. \end{array}$$

In addition, ZF detection projects the received signal y(n) into a space orthogonal to the one spanned by channel vectors of other nodes so that GN *m*’s offloaded symbols can be decoded without inter-stream interference [54].

### 5.3. Action Space

According to the state $s_{m,n}$, each GN *m* will independently select an action $a_{m,n}$ which includes block interval $I_{m}\left(n\right)$, the allocated power for local computing $p_{l,m}\left(n\right)$ and the allocated power for computation offloading $p_{o,m}\left(n\right)$. Consequently, the action space in decision epoch *n* can be defined as:

(19) $$a_{m,n}=\left[p_{l,m}\left(n\right),p_{o,m}\left(n\right),I_{m}\left(n\right)\right],$$

where we have $p_{l,m}\left(n\right)\in \left[0,{\dot{P}}_{l}\right]$, $p_{o,m}\left(n\right)\in \left[0,{\dot{P}}_{o}\right]$ and $I_{m}\left(n\right)\in \left[0,\dot{I}\right]$. Note that the output action of the DDPG algorithm directly maps the states to the optimal power allocation and block generation policy in a continuous action space, which is different from other conventional DRL algorithms where the output is the probability distribution across a discrete action space. Therefore, the dimensional disaster can be avoided in the DDPG algorithm [53].

### 5.4. Reward Function

Considering that each node agent’s behavior is incentive driven, the reward function is important to the convergence of DDPG algorithm. According to the objective of our joint performance optimization problem defined in (16), we construct the reward function $r_{m,n}$ which GN *m* receives after decision epoch *n* as:

(20) $$r_{m,n}=\left\{\begin{array}{cc} \omega \Xi \left(n\right)+\left(1-\omega \right)\xi \sum_{m=1}^{M}\nu_{m}\left(n\right), & \mathrm{if}\;C1-C2\;\mathrm{are}\;\mathrm{satisfied}\\ 0, & \mathrm{otherwise} \end{array}\right.$$

Furthermore, note that the value function of node agent *m* starting from a random initial state $s_{m,1}$ under the policy $\mu_{m}$ can be expressed by

(21) $$\begin{array}{cc} V_{\mu_{m}}\left(s_{m,1}\right) & =E_{\mu_{m}}\left[\sum_{n=1}^{\infty }\gamma^{n-1}r_{m,n}|s_{m,1}\right]\\ \; & =E_{\mu_{m}}\left[r_{m,n}+\gamma^{n-1}\cdot V^{\mu_{m}}\left(s_{m,n+1}\right)|s_{m,1}\right], \end{array}$$

where γ∈[0,1] is the discounting factor in the Bellman equation. The value function $V_{\mu_{m}}$ can be used to quantify the performance of the policy $\mu_{m}$ via an infinite horizon and discounted MDP [55] at node agent *m*. The following average transactional throughput

(22) $${\bar{O}}_{m}\left(s_{m,n}\right)=E\left[\lim_{T\to \infty }\frac{1}{T}\sum_{i=t}^{T}r_{m,i}|s_{m,n}\right],$$

will be maximized by implementing the optimal block generation and computation offloading policy $\mu_{m}^{*}$.

### 5.5. DDPG-Based Algorithm Design

To solve this problem, we provide a model-free and DRL-based approach for finding the optimal block generation and computation offloading strategies jointly. Moreover, owing to the continuous action space of our MDP model, which is intractable by using traditional learning methods, we suggest a DDPG-based approach to address this problem. The DDPG algorithm is a widely used model-free and off-policy algorithm for continuous action spaces. This subsection first introduces the basic mechanism of the DDPG algorithm and then describes the approach to solve the considered problem.

#### 5.5.1. DDPG Background

**Figure 5:**
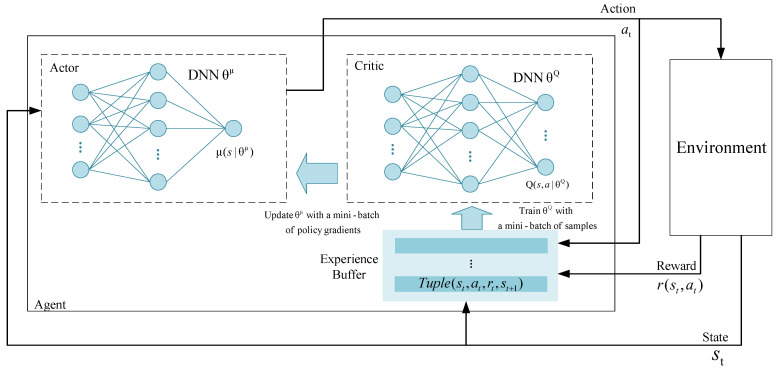
The DDPG algorithm: a model-free, off-policy, and actor–critic algorithm.

As shown in Figure 5, DDPG is a DRL framework that includes two main networks: (1) the actor network and (2) the critic network. Specifically, the actor network is trained for generating the current policy, whereas the critic network is trained for evaluating the advantages and disadvantages of the current policy.

Specifically, the critic network uses neural networks to simulate real *Q*-table to circumvent the curse of dimensionality. Given the current state $s_{n}$, the action $a_{n}$ and the deterministic policy μ, we can write the action-value function as:

(23) $$Q^{\mu }\left(s_{n},a_{n}\right)=E_{s_{n+1},r_{n}∼\Psi }\left[r\left(s_{n},a_{n}\right)+\gamma Q^{\mu }\left(s_{n+1},a_{n+1}\right)\right]$$

where Ψ represents the expectation distribution for $r_{n}$ and $s_{n+1}$.

Similar to the DQN algorithm [26], the critic function $Q(s_{n},a_{n}|\theta^{Q})$ is updated by minimization of the loss function that can be written by:

(24) $$L\left(\theta^{Q}\right)=E_{s_{n}∼\psi^{s},a_{n}∼\psi^{a},r_{n}∼\Psi }\left[{\left(Q\left(s_{n},a_{n}|\theta^{Q}\right)-\epsilon_{n}\right)}^{2}\right]$$

where $\psi^{s}$ and $\psi^{a}$ represent the distribution of state $s_{n}$ and action $a_{n}$, respectively. $\epsilon_{n}$ is given by:

(25) $$\epsilon_{n}=r_{n}+\gamma Q\left(s_{n+1},\mu^{\prime }\left(s_{n+1}\right)|\theta^{Q}\right),$$

where $\mu^{\prime }$ represents the deterministic policy at epoch n+1.

The actor function $\mu (s|\theta^{\mu })$ can map a state *s* to a deterministic action *a* in a continuous space. Based on the critic function, the policy’s updating gradient of the actor is calculated using the chain rule:

(26) $$\nabla_{\theta^{\mu }}J=E_{s_{n}∼\psi^{s}}\left[\nabla_{a}Q\left(s_{n},a|\theta^{Q}\right){|}_{a=\mu (s_{n})}\nabla_{\theta^{\mu }}\mu \left(s_{n}|\theta^{\mu }\right)\right].$$

Therefore, based on (24) and (26), the actor and critic networks’ parameters are softly updated in terms of $\theta^{\mu^{\prime }}\leftarrow \tau \theta^{\mu }+\left(1-\tau \right)\theta^{\mu^{\prime }}$ and $\theta^{Q^{\prime }}\leftarrow \tau \theta^{Q}+\left(1-\tau \right)\theta^{Q^{\prime }}$ where τ is the soft update rate.

#### 5.5.2. The Proposed Algorithm

The detailed DDPG-based optimization algorithm is demonstrated in Algorithm 1 which is implemented in Tensorflow [56]. The algorithm terminates after a preset maximal number of steps $T_{max}$ every episode. For each training episode, the beginning state $s_{m,1}$ is initialized randomly. For each decision epoch *n*, each GN will accumulate and preserve a transition $\left(s_{m,n},a_{m,n},r_{m,n},s_{m,n+1}\right)$ into its own experience memory $B_{m}$. Meanwhile, a random sample of *Z* transitions ${\left\{\left(s_{z},a_{z},r_{z},s_{z}^{{}^{\prime }}\right)\right\}}_{z=1}^{Z}$ from $B_{m}$ will be utilized to update the node’s own actor and critic networks. After the predefined maximum episodes $K_{max}$, each GN will autonomously learn the resource-aware adaptive block generation and computation offloading policy.

Furthermore, at the testing phase, each node agent will directly load the model learned during the previous training phase, and then interact with the environment, beginning with an empty data buffer $U_{m}\left(0\right)$. Similarly, its current state is determined by local observations of the environment and the corresponding action is selected in terms of the output of the actor network.
**Algorithm 1** DDPG-based Optimization Framework for MEC-enabled Blockchain IoT Systems.1:**for** each GN $m\in \Phi_{G}$ **do**2:   **Initialization:** replay memory $B_{m}$, critic network $Q(s,a|\theta_{m}^{Q})$, actor network $\mu (s|\theta_{m}^{\mu })$ and corresponding target networks $Q^{\prime }$ and $\mu^{\prime }$ with weights $\theta_{m}^{\mu^{\prime }}\leftarrow \theta_{m}^{\mu }$ and $\theta_{m}^{Q^{\prime }}\leftarrow \theta_{m}^{Q}$;3:**end for**4:**for** each episode $\in \{1,2,\ldots ,K_{max}\}$ **do**5:   **Initialization:** state $s_{m,1}$ for each GN $m\in \Phi_{G}$;6:   **for** each decision epoch $n=1,2,\ldots ,T_{max}$ **do**7:     **for** each GN $m\in \Phi_{G}$ **do**8:        Select action $a_{m,n}=\mu \left(s_{m,n}|\theta_{m}^{\mu }\right)+\Delta \mu$ based on the exploration noise Δμ to decide block interval and power allocation;9:        Observe reward $r_{m,n}$ and next state $s_{m,n+1}$;10:        Store transition data $(s_{m,n},a_{m,n},r_{m,n},$ $s_{m,n+1})$ into replay memory $B_{m}$;11:        Sample a mini-batch of *Z* transition tuples ${\left\{\left(s_{z},a_{z},r_{z},s_{z}^{\prime }\right)\right\}}_{z=1}^{Z}$ from memory $B_{m}$ at random;12:        Update critic network by minimizing the loss *L*:
$L\;=\frac{1}{Z}\sum_{z=1}^{Z}{\left(Q\left(s_{z},a_{z}|\theta_{m}^{Q}\right)-\epsilon_{z}\right)}^{2};$13:        Update actor policy based on the sampled policy gradient:
$\nabla_{\theta_{m}^{\mu }}J\approx \frac{1}{Z}\sum_{z=1}^{Z}\nabla_{a}Q\left(s_{z},a|\theta_{m}^{Q}\right){|}_{a=\mu (s_{z})}\nabla_{\theta_{m}^{\mu }}\mu \left(s_{z}|\theta_{m}^{\mu }\right);$14:        Update target networks:
$$\theta_{m}^{\mu^{\prime }}\leftarrow \zeta \theta_{m}^{\mu }+\left(1-\zeta \right)\theta_{m}^{\mu^{\prime }}$$
$\theta_{m}^{Q^{\prime }}\leftarrow \zeta \theta_{m}^{Q}+\left(1-\zeta \right)\theta_{m}^{Q^{\prime }}$15:     **end for**16:   **end for**17:**end for**

## 6. Simulation Results and Discussions

We compare the proposed distributed DDPG-based scheme to some other baseline schemes in different simulation scenarios in this part. Additionally, simulation results are provided to demonstrate the proposed DDPG-based framework for performance optimization in the MEC-enabled blockchain IoT system. For the software environment, all code is implemented in Tensorflow Version 1.15.0 with Python 3.7 in a Windows 10 system. The main simulation parameters are summarized in Table 3.

### 6.1. Simulation Setup

At the start of each episode, each GN m’s channel vector can be predefined as $h_{m}\left(0\right)∼CN\left(0,h_{0}{\left(d_{0}/d_{m}\right)}^{\alpha }I_{N_{a}}\right)$. Specifically, $h_{0}$, $d_{0}$, α, and $d_{m}$ denote path-loss constant, reference distance, path-loss exponent, and distance between GN *m* and BS, respectively. During the following decision epochs, $h_{m}\left(n\right)$ is updated in terms of (2) [44].

For the implementation of the DDPG algorithm, we have an actor network learning the policy network approximation $\mu (s|\theta_{m}^{\mu })$ and a critic network predicting the Q-function network approximation $Q(s,a|\theta_{m}^{Q})$ concurrently. Specifically, the DDPG algorithm is constructed with a fully connected neural network that includes one input layer, two hidden layers, and one output layer. In our simulations, we assume that there are 400 and 300 hidden neurons in these 2 hidden layers, respectively. In addition, the activation function for the two hidden layers is chosen to be f(x)=max(0,x), and a sigmoid layer is used to bound the output actions. The discounting factor γ=0.99 is used. Moreover, we utilize the adaptive moment estimation (Adam) method to learn the neural network parameters, and meanwhile, the learning rates for the actor and critic networks are set as shown in Table 3 [57]. Similar to [53], the final output layer weights and bias of both the actor network and critic network are initialized from a uniform distribution [−0.003,0.003] and [−0.0003,0.0003], while the layer weights of other layers are based on the fan-in of the layer. We adopt the Ornstein–Uhlenbeck process (θ=0.15, σ=0.15) to introduce the temporally correlated noise to explore the action space more efficiently [58].

For comparison, three baseline schemes are introduced in the simulation section:

(1) *Greedy local-execution-first scheme (GD-Local):* For each decision epoch *n*, each node agent *m* attempts to execute buffered data bits locally first, and then offloads the remainder of computation tasks to the MEC server.
(2) *Greedy computation-offloading-first scheme (GD-Offload):* For each decision epoch *n*, the node agent *m* attempts to offload buffered task bits to the MEC server first, and then the remaining computation tasks are processed locally.
(3) *DQN-based dynamic offloading scheme (DQN):* DQN is a DRL algorithm with a discrete action space [26]. Specifically, for each node agent *m*, we tune the power allocated for local computing and computation offloading from limited candidate sets $P_{l,m}=\left\{0,\frac{{\dot{P}}_{l}}{L-1},\ldots ,{\dot{P}}_{l}\right\}$ and $P_{o,m}=\left\{0,\frac{{\dot{P}}_{o}}{L-1},\ldots ,{\dot{P}}_{o}\right\}$, respectively, in which L denotes the total number of discrete levels.

### 6.2. Performance of the Proposed Scheme

**Figure 6:**
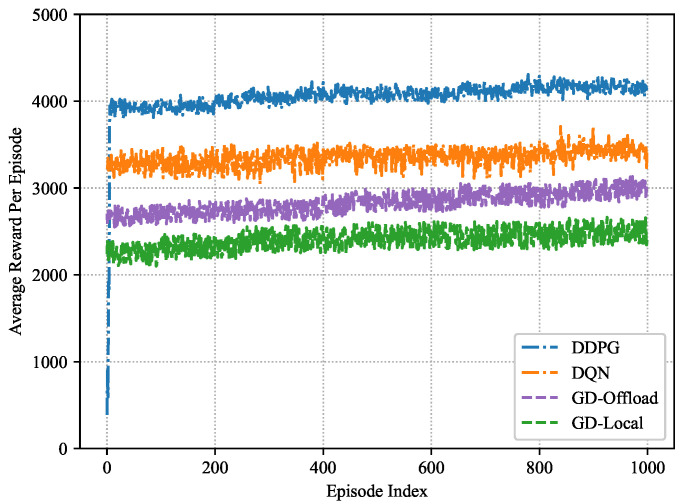
Convergence performance based on different schemes in the training process.

As seen in Figure 6, the convergence performance of the proposed DDPG-based performance optimization scheme and three baseline schemes is presented, respectively. In Figure 6, as the number of episodes increases, the average reward increases as well. The average reward becomes stable after about 800 episodes, which verifies that effective performance optimization strategies can be learned without prior knowledge. In addition, the DDPG-based scheme can achieve about 92.86% of the optimal performance after training 100 episodes. Guo, F. et al. [32] showed that the double-dueling DQN-based scheme required 60 episodes of training to achieve 90.48% of the optimal performance. Feng, J. et al. [59] shows that the A3C-based scheme achieves about 91.86% of the optimal performance after training 150 episodes. Therefore, the DDPG-based scheme does not lead to more system overhead than other schemes.

For Figure 6, it is worth noting that DRL-based schemes outperform both greedy schemes as they cannot dynamically and adaptively allocate computing resources, which verifies the superiority of DRL-based schemes. Even though the DQN-based scheme enables the performance of MEC and blockchain systems in IoT networks to be optimized concurrently, the policy learned by the DQN-based scheme may not be the global optimum as the spaces of power allocation and block interval are continuous. However, the DDPG-based scheme with a continuous action space enables discovering the globally optimum policy. It can be found that the performance of the strategy derived from the DDPG-based scheme is superior to the other three baselines, demonstrating the benefit of our suggested framework for continuous control problems.

**Figure 7:**
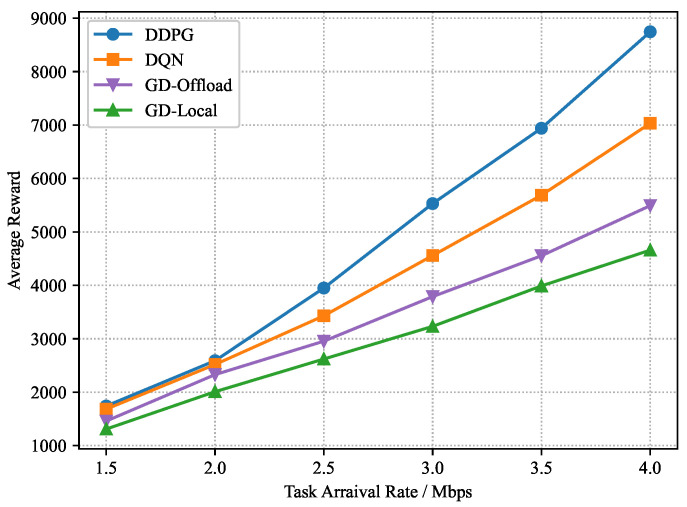
Average reward versus task arrival rate.

**Figure 8:**
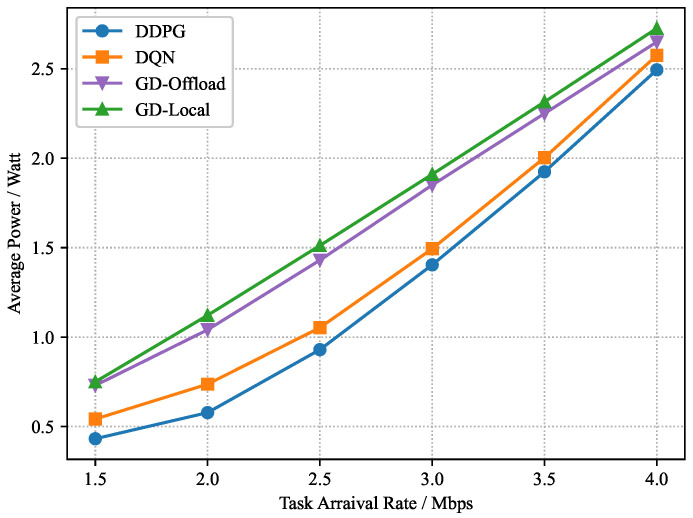
Average power consumption versus task arrival rate.

**Figure 9:**
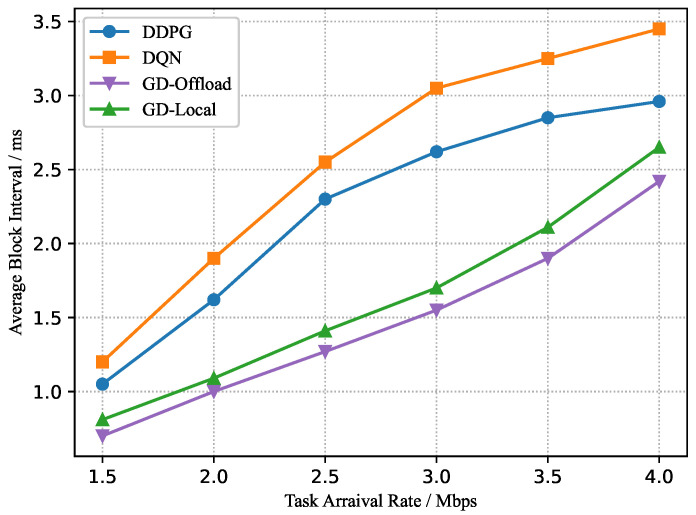
Average block interval versus task arrival rate.

In the training process, the parameters of the critic and actor networks are updated continuously. After $K_{max}=1000$ episodes, we can obtain the trained *c* for the dynamic block generation and computation offloading in the testing process. Considering a set of task arrival rates $\lambda_{m}=1.5∼4.0$ Mbps, we train these four considered schemes independently with the same system parameters for different task arrival rates. As shown in Figure 7, Figure 8 and Figure 9, as the task arrival rate increases, all schemes achieve a greater average reward, implying that a larger computation demand requires a larger computation cost. Meanwhile, due to the growing computation cost, the average power consumption and buffering delay increase progressively. Furthermore, the suggested DDPG-based scheme can consistently achieve better average rewards than other schemes, which implies that higher blockchain system throughput and MEC computational efficiency can be obtained under the DDPG-based scheme. Moreover, from Figure 9, it is noted that the DDPG-based scheme slightly compromises the average block interval to obtain the lowest power consumption.

**Figure 10:**
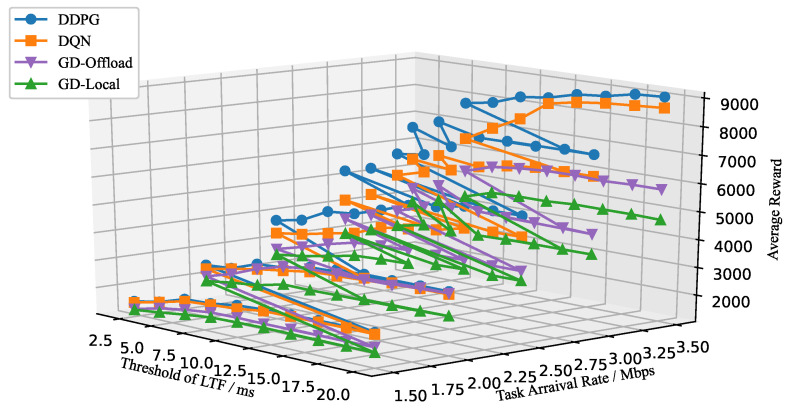
Average reward versus threshold of LTF and task arrival rate.

**Figure 11:**
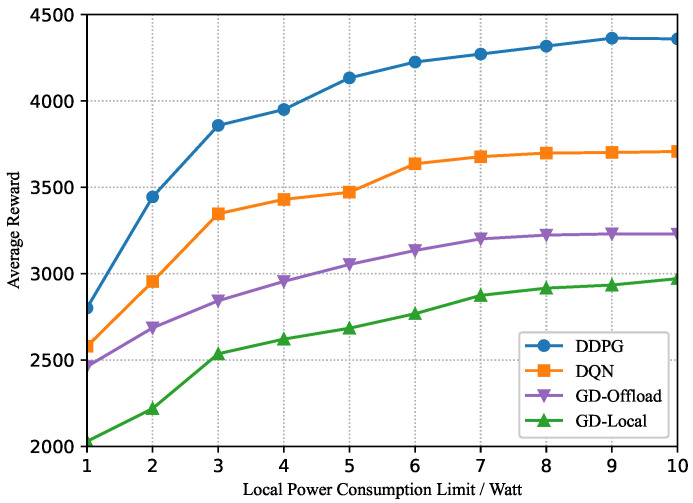
Average reward versus threshold of local power consumption.

Furthermore, we analyze the testing results of the MEC-enabled blockchain system by setting different threshold values of LTF or local power consumption. Specifically, the effect of the block interval limit is depicted in Figure 10. It has been shown that the average reward increases gradually when the threshold of LTF and task arrival rate grow. An explanation is that each node can process more data transactions over one block under a more relaxed latency constraint. However, at a fixed task arrival rate, the long-term reward value increases and reaches a stable state as the threshold of LTF increases, because the allocable time gradually becomes abundant. As the task arrival rate gets larger, the time to reach saturation is gradually delayed. Moreover, we can see that the DDPG-based scheme can consistently achieve a much higher average reward than the greedy and DQN-based schemes. Figure 11 examines the average reward with different thresholds of local power consumption ${\dot{p}}_{m}={\dot{p}}_{o,m}+{\dot{p}}_{l,m}$. Additionally, one observation is that the MEC-enabled blockchain system can process more data transactions when the threshold of the local power consumption increases. However, the average reward does not grow infinitely in Figure 11, because the latency constraint and task arrival rate restrict the maximum amount of data transactions in one block. These numerical results can help with the design of the MEC-enabled blockchain systems in real-world situations.

**Figure 12:**
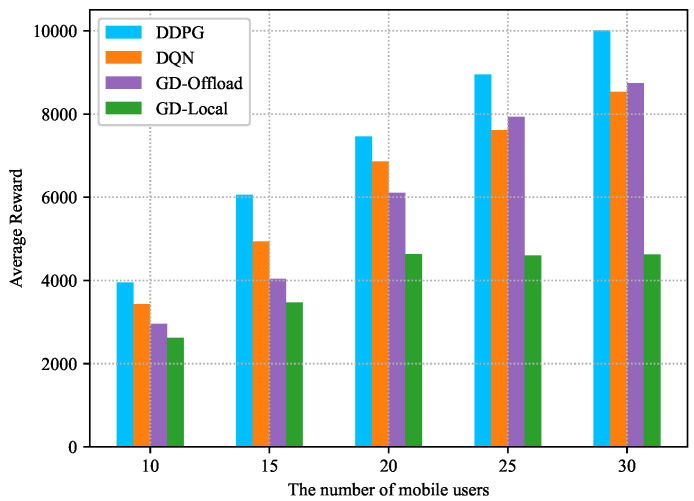
Average reward versus the number of mobile users.

Figure 12 illustrates the variation of the average reward as the number of nodes increases, demonstrating the scalability of the proposed framework and its ability to adapt to dynamic changes in the user profile. As the number of participating nodes grows, the number of data transactions that need to be packaged increases, and thus the average reward gradually increases. Furthermore, as the number of mobile users increases, the superiority of the DDPG-based scheme over other schemes grows and the performance GD-local scheme declines after the number exceeds 20. Therefore, as long as the size of the computational task is within the processing power of the MEC server, the size of the block generated and validated after one epoch also becomes larger, and meanwhile, the LTF constraint can be satisfied. Based on the proposed joint optimization scheme of local execution power, computation offloading power, block size, and block interval, the DDPG-based algorithm can achieve a better performance than the other considered schemes.

**Figure 13:**
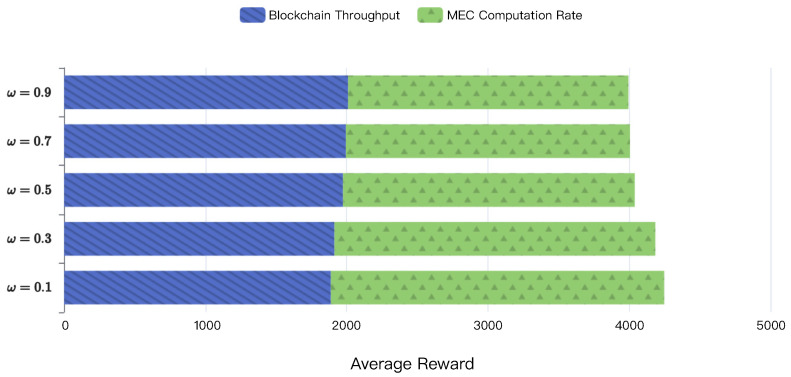
The effect of different weight factors on average reward.

Figure 13 shows the effect of different weight factors on the average reward. We can observe that the overall average reward slightly increases as the weight factor ω decreases. That is because the overall performance of the considered framework mainly depends on the MEC computation rate, and a smaller weight factor indicates a greater emphasis on MEC optimization. The simulation performance of four schemes under different parameters is summarized in Table 4.

## 7. Conclusions

In this paper, we studied an MEC-enabled blockchain system for future wireless IoT networks and investigated the joint performance optimization problem of blockchain transaction throughput and MEC computational efficiency. The joint problem of MEC computation offloading and block generation policy was formulated, where the power allocation and block interval were optimized. As a result of the time-varying properties of wireless channels in this system, we modeled the joint optimization problem as an MDP. A DDPG-based algorithm was proposed to solve the MDP problem, which can cope with the problem under continuous action space and learn the optimal strategy without prior knowledge of the environment.

The simulation results have demonstrated the proposed scheme outperforms DQN-based and some other greedy (non-joint optimization) schemes under different task arrival rates, thresholds of LTF, and weight factors. The joint optimization scheme can achieve better performance than other schemes with a high convergence rate. Meanwhile, we evaluated its performance in terms of power consumption and block interval, and the simulation results have shown that the joint optimization scheme slightly compromises the average block interval to obtain the lowest power consumption. Additionally, we discussed the impact of the number of mobile users on the convergence performance and the joint optimization scheme always has an advantage over other schemes.

In conclusion, the proposed scheme paves the road for the efficient deployment of blockchain technology in IoT networks, which can be applied in latency-sensitive and security-sensitive IoT applications (e.g., internet of vehicles, virtual reality, smart healthcare, etc.).

## Figures and Tables

**Table 1:** A comparison with related works.

Work	Long-Term Reward	Joint Optimization	Blockchain Agent	State Space Design	ML Approach
[21]	×	×	×	/	×
[22]	✓	×	×	A	DQN
[31]	✓	×	IoT Community	B	AGA
[32]	✓	✓	Base Station (BS)	C	Double-Dueling DQN
Our proposed scheme	✓	✓	IoT Device	D	DDPG

**Table 2:** Notation definitions.

Notation	Description
$\Phi_{G}$	The set of GNs.
$\Phi_{V}$	The set of VNs.
*M*	The number of GNs.
*K*	The number of VNs.
$L_{n}$	The number of time slots included in decision epoch *n*.
$h_{m}\left(\tau \right)$	Channel vector between GN *m* and the BS at time slot τ.
y(τ)	Received signal of the BS at time slot τ.
$\rho_{m}$	Normalized temporal channel correlation coefficient of GN *m*.
H(τ)	Channel matrix from all the nodes to the BS at time slot τ.
$\gamma_{m}\left(\tau \right)$	The receiving SINR of GN *m* at time slot τ.
$\phi_{m}\left(n\right)$	ZF detection vector for GN *m* at time slot τ.
$\lambda_{m}$	Task arrival rate of GN *m*.
$U_{m}\left(n\right)$	Queue length of GN *m*’s task buffer at decision epoch *n*.
$a_{m}\left(n\right)$	Number of task arrivals of GN *m* at decision epoch *n*.
$f_{m}\left(\tau \right)$	CPU frequency scheduled for local execution of GN *m* at time slot τ.
$C_{m}\left(F_{m}\right)$	CPU cycles required per one task bit (allowable CPU-cycle frequency) at GN *m*.
$p_{o,m}\left(n\right)$	Transmission power of GN *m* for computation offloading at decision epoch *n*.
$d_{o,m}\left(\tau \right)\left(D_{o,m}\left(n\right)\right)$	Data transmitted by GN *m* for computation offloading at time slot τ (decision epoch *n*).
$p_{l,m}\left(n\right)$	Power consumption of GN *m* for local execution at decision epoch *n*.
$d_{l,m}\left(\tau \right)\left(D_{l,m}\left(n\right)\right)$	Data processed by GN *m* via local execution at time slot τ (decision epoch *n*).
$P_{o,m}\left(P_{l,m}\right)$	Maximum transmission power (local execution power) of GN *m*.
$\nu_{m}\left(\tau \right)$	Computation rate of GN *m* during time slot τ.
Y(n)	The set of stakes IoT nodes hold at decision epoch *n*.
$I_{m}\left(n\right)\left(I_{P}\left(n\right)\right)$	Block interval of GN *m* (PN) at decision epoch *n*.
$T_{F}\left(n\right)$	Latency time to finality at decision epoch *n*.
$S^{B}\left(n\right)$	Block size at decision epoch *n*.
Ξ(n)	Blockchain transaction throughput at decision epoch *n*.

**Table 3:** Simulation parameters.

Parameter	Value
The number of action levels in DQN, L	8
The path-loss constant in channel vector, $h_{0}$	−30 dB
The reference distance in channel vector, $d_{0}$	1 m
The length of one time slot, $\tau_{0}$	10 ms
The pass-loss exponent in channel vector, α	3
The channel correlation coefficient, $\rho_{m}$	0.95
The Doppler frequency of GN *m*, $f_{m}^{d}$	70 Hz
System bandwidth, *W*	1 MHz
Average transaction size, χ	200 B
The maximum transmission power, ${\dot{P}}_{o}$	4 W
The maximum power available for local execution, ${\dot{P}}_{l}$	4 W
The maximum number of time slots for one epoch, $\dot{L_{n}}$	20
The noise power, $\sigma_{R}^{2}$	$10^{-9}$ W
The effective switched capacitance, ι	$10^{-27}$
The required CPU cycles for each task bit, $C_{m}$	500 cycles/bit
The maximum allowable CPU-cycle frequency, $F_{m}$	1.26 GHz
The weight factor, ω	0.5
The soft update rate for the target networks, ζ	0.001
The number of episodes, $K_{max}$	1000
The maximum steps of each episode, $T_{max}$	1000
The task arrival rate, $\lambda_{m}$	2.5 Mbps
The thresholds of decentralization, $\eta_{s},\eta_{l}$	0.2, 0.3

**Table 4:** Performance comparison in terms of average reward.

		DDPG	DQN	GD-Offload	GD-Local
*Traning* episodes	*100*	3856.02	3381.07	2784.23	2371.89
	*500*	4156.14	3414.74	2885.67	2357.42
	*1000*	4177.97	3445.40	3012.81	2519.65
*Task arrival rate/Mbps*	*1.5*	1742.25	1683.47	1460.23	1310.20
	*2.5*	3950.14	3430.28	2955.37	2621.09
	*3.5*	6940.87	5687.55	4554.54	3989.32
*Threshold of LTF/ms*	*2.0*	3761.19	3289.24	2691.85	2478.72
	*7.5*	4443.35	3644.41	3250.13	2799.44
	*12.5*	4857.52	4228.17	3569.60	3070.02
*Local power consumption limit/watt*	*2*	3444.58	2954.47	2686.43	2219.36
	*5*	4133.25	3471.36	3053.31	2684.68
	*8*	4317.63	3698.01	3223.30	2916.34
*The number of mobile users*	*10*	3950.56	3430.09	2955.40	2621.34
	*20*	7461.89	6862.15	6110.04	4630.71
	*30*	10,006.53	8553.49	8742.11	4621.98

## Data Availability

Not applicable.

## Abbreviations

The following abbreviations are used in this manuscript:

BS	Base Station
CPU	Central Processing Unit
DDPG	Deep Deterministic Policy Gradient
DPoS	Delegated Proof-of-Stake
DQN	Deep Q Network
DRL	Deep Reinforcement Learning
GN	General Node
IoT	Internet of Things
LTF	Latency Time to Finality
MAC	Message Authentication Code
MDP	Markov Decision Process
MEC	Mobile Edge Computing
PBFT	Practical Byzantine Fault Tolerance
PN	Primary Node
PoS	Proof of Stake
PoW	Proof of Work
SINR	Signal to Interference-plus-Noise Ratio
VN	Validation Node
ZF	Zero Forcing
